# Stay Connected to Be Diverse!

**DOI:** 10.1111/gcb.70046

**Published:** 2025-01-23

**Authors:** Maria Stockenreiter

**Affiliations:** ^1^ Aquatic Ecology, Department Biology Ludwig‐Maximilians – University Munich München Germany

**Keywords:** aquatic ecosystems, climate change impacts, connectivity loss, mesocosms, phytoplankton diversity

## Abstract

Plankton biodiversity is crucial for the functioning of aquatic ecosystems, influencing nutrient cycling, food web dynamics, and carbon storage. Global change and habitat destruction disrupt these ecosystems, reducing species diversity and ecosystem resilience. Connectivity between aquatic habitats supports biodiversity by enabling species migration, genetic diversity, and recovery from disturbances. However, research on how connectivity loss impacts plankton remains limited. A study by Szabó et al. used controlled experiments to show that habitat fragmentation significantly influences phytoplankton diversity. These findings highlight the need to conserve both biodiversity and habitat connectivity to sustain freshwater ecosystems and combat global environmental challenges.

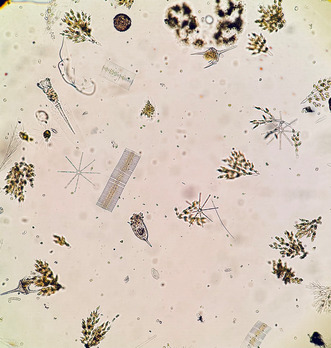

Biodiversity in plankton communities is critical for the functioning of aquatic ecosystems, and its significance is intensified under the pressures of global change. Plankton, comprising primary producers (phytoplankton) and consumers (zooplankton), are central to nutrient cycling, food web dynamics, and carbon sequestration in aquatic ecosystems. As climate change, pollution, and habitat alteration increasingly disrupt aquatic environments, preserving plankton biodiversity is essential for ecosystem resilience (Downing and Leibold 2010). However, global changes lead to shifts in abiotic and biotic factors impacting plankton species composition and abundance. These changes may favor certain species, such as harmful phytoplankton, over others, reducing ecosystem stability, and altering food webs. For instance, reduced phytoplankton diversity lowers primary productivity, affecting food chains and diminishing ecosystems' carbon capture potential (Henson et al. 2021). Similarly, disruptions in zooplankton populations impair nutrient cycling and predator–prey interactions (Downing and Leibold 2010). Biodiversity serves as a buffer, enabling plankton communities to adapt to environmental changes and maintain ecosystem functions. A diverse community is more likely to include species that tolerate stressors, enhancing ecosystem resilience through complex food webs and effective nutrient cycling (Yachi and Loreau 1999). Consequently, conserving plankton biodiversity is vital for mitigating global environmental changes.

Habitat destruction is a major driver of biodiversity loss, including the decline of plankton and other aquatic species. In aquatic ecosystems, disruptions lead to the loss of important connections among different water bodies, which can result in destabilized ecosystems. The connectivity among freshwater systems is critical for plankton biodiversity, supporting ecosystem health, productivity, and food web stability (D'Alelio et al. 2016). It fosters metacommunity structures, where local plankton populations interact via dispersal, stabilizing overall biodiversity (Guelzow et al. 2017). Connected systems facilitate gene flow among populations, enhancing genetic diversity, and enabling plankton to adapt to environmental changes. High genetic diversity reduces the risk of population collapse due to diseases or environmental stress. Moreover, connected freshwater systems allow plankton species to migrate in response to changing conditions, such as warming temperatures or nutrient imbalances. In fragmented habitats, connectivity accelerates recolonization and recovery. Already Darwin noted passive transport mechanisms, such as aquatic organisms being carried by birds, while early ecologists like Naumann and Kretschmar (1955) explored connectivity's role in nutrient and sediment transport. Later frameworks, such as the theory of island biogeography and metacommunity theory (Leibold 2011), emphasized connectivity as pivotal for species persistence and biodiversity patterns. The role of connectivity has evolved from being a peripheral concept to a cornerstone in understanding how plankton communities are structured. This historical trajectory underscores connectivity as vital for maintaining the biodiversity and resilience of freshwater plankton communities.

The surprising thing is that experimental research on the effect of loss of connectivity on microbial communities is rare. Salis, Brennan, and Hansson (2023) investigated the success of invasive phytoplankton species under different dispersal and increased water temperature conditions. With their study, they were also able to give an estimate for altered biodiversity in their phytoplankton communities; however, it was not the main goal of their study to mechanistically find out the effect of connectivity per se on biodiversity. Studies often use observational or survey data to infer the impact of connectivity on biodiversity in natural settings (Vanormelingen et al. 2008). In aquatic systems, connectivity research frequently targets zooplankton or larger aquatic organisms rather than phytoplankton or often uses modeling frameworks (e.g., metacommunity theory) (Cloern 2007). Those models aim to predict the effects of habitat fragmentation and connectivity loss, but they lack direct experimental validation, especially for phytoplankton. Why there are so few experimental studies in natural communities? Simulating natural connectivity loss and fragmentation in controlled experimental setups is challenging, and often studies are carried out on a laboratory scale, which might lack representing natural settings enough. Mesocosm experiments, however, are valuable tools in ecological and environmental research for several reasons. These experiments involve creating controlled, semi‐natural environments that bridge the gap between laboratory studies and field research and are well established in aquatic research.

The study by Szabó et al. (2024) highlighted in *Global Change Biology* uses mesocosm experiments (Figure 1) to investigate the role of connectivity loss in filling the aforementioned gap of knowledge for the very important basis of almost all aquatic food webs—phytoplankton. By experimentally disconnecting previously connected habitats, the study effectively simulates real‐world fragmentation. This approach minimizes external biases and provides a realistic assessment of how connectivity loss impacts biodiversity.

**FIGURE 1:**
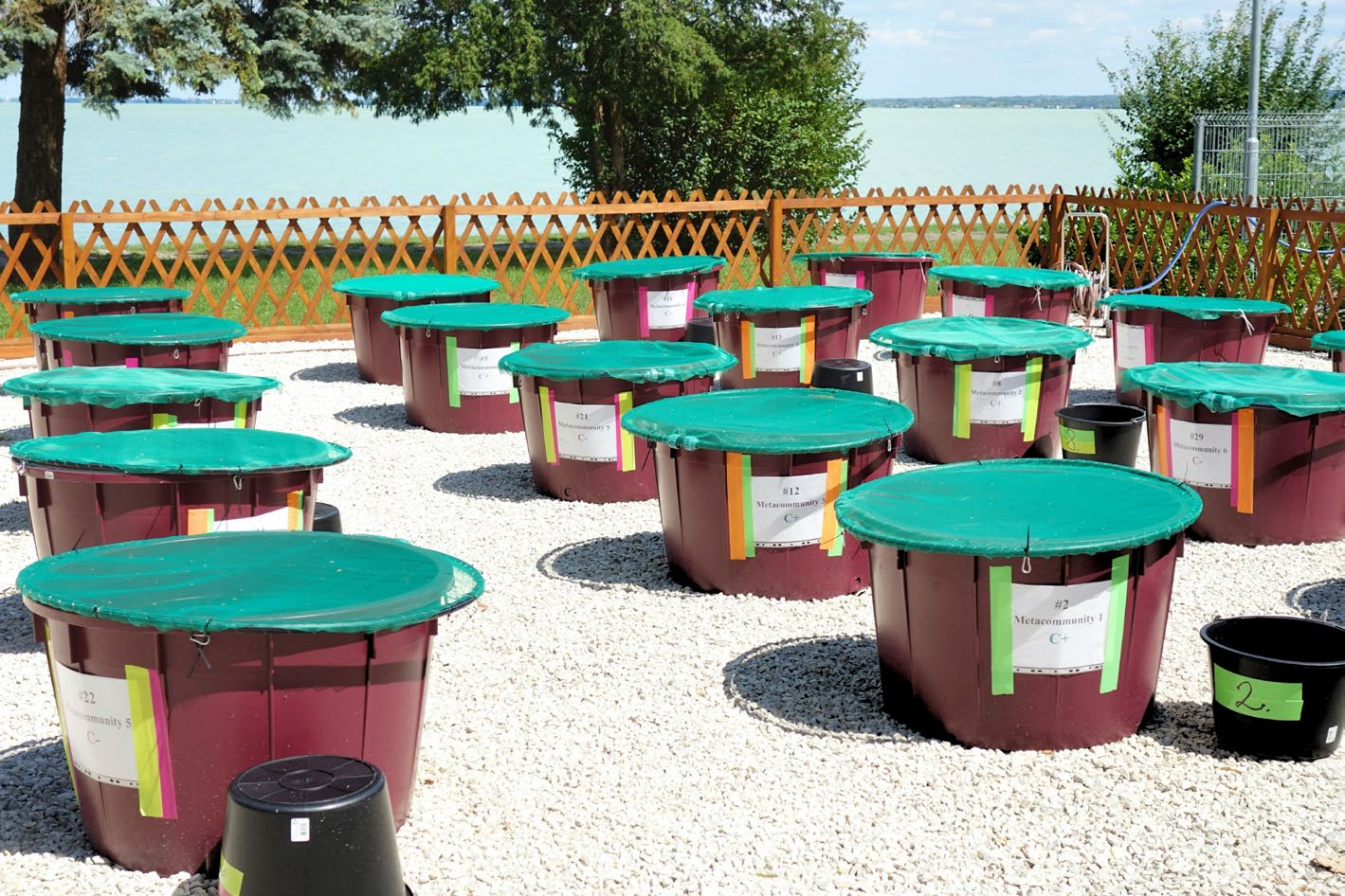
Experimental setup of the mesocosm study regarding the effects of connectivity loss on phytoplankton communities. Photo credits: Beáta Szabó. *Storage*: Global change and habitat destruction disrupt these ecosystems, reducing species diversity and ecosystem resilience. Connectivity between aquatic habitats supports biodiversity by enabling species migration, genetic diversity, and recovery from disturbances. However, research on how connectivity loss impacts plankton remains limited. A study by Szabó et al. used controlled experiments to show that habitat fragmentation significantly influences phytoplankton diversity. These findings highlight the need to conserve both biodiversity and habitat connectivity to sustain freshwater ecosystems and combat global environmental challenges.

Szabó et al.'s study in *Global Change Biology* provides valuable insights into how connectivity loss impacts diversity and explores the effects of fragmentation on local and regional biodiversity, focusing on phytoplankton communities. In their mesocosm study, they found that connectivity loss significantly reduces both local and regional biodiversity, especially in eukaryotic phytoplankton. Fragmentation decreases species richness and evenness, indicating that it disproportionately affects both rare and common taxa. Surprisingly, the prokaryotic phytoplankton are less affected by connectivity mainly due to their smaller size, larger population sizes, and higher rate of propagule rates (De Bie et al. 2012). These findings emphasize the role of phytoplankton traits in determining fragmentation sensitivity. The study identifies grazer biomass, such as zooplankton, as a key factor influencing phytoplankton biodiversity in fragmented habitats. Changes in grazer populations alter the phytoplankton community structure (e.g., reduced evenness), linking food web interactions with biodiversity patterns (e.g., ratios in eukaryotic to prokaryotic phytoplankton). Fragmentation may lead to extinction debts, particularly in prokaryotic communities. Species with low regional abundance exhibit delayed declines, suggesting that long‐term studies are necessary to fully understand the effects of fragmentation. The findings provided by Szabó et al. highlight the complexity of fragmentation and connectivity loss, particularly for microbial communities. They also underscore the importance of maintaining connectivity to support biodiversity and ecosystem stability. In the face of ongoing global challenges, conserving both biodiversity and connectivity is imperative for ensuring the health and sustainability of freshwater ecosystems. These efforts are critical not only for aquatic organisms but for the broader environmental and ecological systems they support.

## Author Contributions

**Maria Stockenreiter:** conceptualization, writing – original draft, writing – review and editing.

## Conflicts of Interest

The author declares no conflicts of interest.

## Data Availability

Data sharing is not applicable for this article as no datasets were generated or analyzed for the current article.
